# The molecular characterization by SSRs reveals a new South Italian kinship and the origin of the cultivar *Uva di Troia*

**DOI:** 10.1186/s40064-016-3228-8

**Published:** 2016-09-14

**Authors:** C. Bergamini, R. Perniola, M. F. Cardone, M. Gasparro, R. Pepe, A. R. Caputo, D. Antonacci

**Affiliations:** 1 Consiglio per la ricerca in agricoltura e l’analisi dell’economia agraria– CREA - Unità di ricerca per l’uva da tavola e la vitivinicoltura in ambiente mediterraneo, Research Unit for Viticulture and Enology in Southern Italy, Via Casamassima, 148, 70010 Turi, BA Italy; 2 Consiglio per la ricerca in agricoltura e l’analisi dell’economia agraria– CREA – Centro di ricerca per l’orticoltura, Via Cavalleggeri, 25, 84098 Pontecagnano, SA Italy

**Keywords:** Grapevine, *Uva di Troia*, *Quagliano*, *Bombino bianco*, *Bombino nero*, *Impigno*, Microsatellites, Kinship

## Abstract

*Vitis vinifera* L. varieties were spread through cuttings following historic migrations of people, trades, or after biological crises due to pests outbreaks. Some today’s varieties could be more than a 1000 years old and, although over the centuries these varieties generated most of the remaining cultivars, their origin could be impossible to track back. The Italian grapevine biodiversity is one of most important, most likely due to its strategic position in the middle of the Mediterranean sea. Unravelling of its structure is challenging because of its complexity and the lack of historical documentation. In this paper molecular data are compared with historical documentations. Simple Sequence Repeats fingerprinting are molecular markers best suited to investigate genetic relationships and identify pedigrees. South-Italian germplasm was studied with 54 nuclear microsatellites. A family was identified, consisting of two parents and three siblings and further genetically characterized with six nuclear and five chloroplast microsatellites and described with ampelographic and phylometric analysis. Although these latter were not informative for the kinship identification. The common *Bombino bianco* was the female parent and the previously unknown *Uva rosa antica* was the male parent. *Bombino nero*, *Impigno* and the popular *Uva di Troia*, all typical of the south-east Italy, were the offspring. Further research showed that the *Uva rosa antica* was a synonym of *Quagliano* and *Bouteillan noir*, both minor varieties. *Quagliano* was considered to be autochthonous of some alpine valleys in the north-west of Italy and *Bouteillan noir* is a neglected variety of Vancluse in France. This finding uncovers the intricate nature of Italian grape cultivars, considered peculiar of an area, but possibly being the remains of ancient latin founding varieties. Consequently, intriguing new hypotheses are discussed and some conclusions are drawn, based on the peculiar geographical origin of the parents, on the distribution of the offspring, on the chance of a single, and perhaps intentional, crossing event.

## Background

*Vitis vinifera* is one of the earliest domesticated crop and it is native of the Mediterranean and Middle East areas. Dioecy and allogamy are found in all wild *Vitis vinifera* L. subsp. *sylvestris* and for a successful reproduction, cross-pollination is required while inbreeding leads to severe genetic depression of progeny. However, in the cultivated species (subspecies *sativa*) hermafroditism and self-pollination were selected during the domestication process to improve berry production. The issues that would arise from this contrast are avoided thanks to the process of vegetative propagation, a procedure that allows to fix favorable genes combinations in selected genotypes, making them virtually immortal and escape the need for sexual reproduction. Consequently, some varieties might be very ancient: *Pinot noir*, *Muscat Blanc à Petits Grains*, and *Sultanina,* for example, date back probably to one or two thousands of years ago (Bowers et al. 1999a; This et al. 2006). Moreover, the population structure of *Vitis vinifera* generated recently a considerable research interest but it is still debated, mostly because it is not clear how accurately germplasm collection used in these studies represent the real population of this species (Myles et al. 2011; Emanuelli et al. 2013; Bacilieri et al. 2013). Indeed, some researchers believe that few varieties are actually progenitors of most of the relevant cultivated genotypes and such elite cultivars are responsible for the complex and close relationships between the cultivars. (Myles et al. 2011).

*Vitis vinifera* growing practice was spread following historic migrations of people and progression of civilization; the same flow might be assumed for cultivated varieties. Many grape varieties have no clear origins, not only for the nineteenth century outbreaks of American diseases that lead to an extinction of many ancient varieties and heavy reduction of diversity, but also because of the shuffling and exchanges of cuttings that occurred over the centuries following trades and migrations, resulting in a masking of the true origins of autochthonous cultivated varieties (McGovern 2004). Likewise, synonymy and homonymy occurrence in grapevine varieties makes origin assessment and pedigree reconstruction even more difficult. In this context, molecular markers could be used to solve these challenges; in particular, fingerprints of microsatellites, also called Simple Sequence Repeats (SSR), can unequivocally identify varieties despite changes of plant phenotype in different environmental conditions and severe changes in morphology due to virus infection and lack of vigour. Likewise, an additional ideal use of SSR fingerprinting is to investigate genetic relations and identify pedigrees, because of the co-dominant, neutral behavior and Mendelian segregation of these markers (Dakin and Avise 2004).

Although SSR fingerprinting has been used by many researchers to investigate the Italian grapevine biodiversity, one of the greatest considering its strategic position in the middle of the Mediterranean Sea, results so far have been challenging to be interpreted (Sefc et al. 2000). The most important autochthonous cultivated varieties appear uncorrelated. As an example, the origins of the renowned variety *Uva di Troia* are still at the mythological tale state, being believed to date back to the arrive in Daunia (Apulia region) of the hero Diomedes, who carried cuttings of grapevines from the Anatolian city of Troy after its destruction, as described by Homer in the Iliad.

In this paper we present a study that started from the characterization of the South Italian grapevine biodiversity by means of 54 nuclear and 5 chloroplast microsatellites, then proceeded to analyze possible parental relationship. This analysis gave surprising results: a whole new family of renowned grapevine varieties, composed by two parents and three siblings. In order to investigate the genealogy of *Uva di Troia*, South Italian grapevine biodiversity has been used with more than 2000 accessions previously screened for genetic relatedness by 13 SSRs (Bergamini et al. 2013). In this study 107 grapevine cultivars best representing South Italian grapevine biodiversity have been genotyped by 54 nuclear and 5 chloroplast microsatellites in order to look for genetic relatedness among them. Also, the study included detailed analysis of similarity in ampelographic characteristics and search of historical backgrounds in case of established kinships.

## Methods

### Plant material

The germplasm collection of CREA-UTV in Turi (Bari, Italy) (Lat. 40°92 57′24.54″N, Lon.17° 00′28.94″E) includes internationally spread varieties of both table and wine grapes and consist of about a thousand of unique genotypes. The collection includes autochthonous varieties of the Southern Italian regions (Basilicata, Calabria, Campania, Apulia and Sicily). A sup-population, consisting of 107 most diffused and cultivated varieties, have been selected as best representing South-Italian grapevine biodiversity and further characterized in this study.

### DNA extraction and molecular characterization

DNA fingerprinting was performed as described in Bergamini et al. (2013). Briefly, genomic DNA was extracted from young leaf tissue using Qiagen DNeasy Plant Mini Kit (Qiagen, Valencia, CA) on liquid nitrogen-frozen leaf samples, after homogenization by Qiagen Tissue Lyser (Qiagen, Valencia, CA), according to the manufacturer instruction protocol. Purified DNA was used as template in a PCR amplification for genotyping using 60 nuclear SSR loci ISV2, ISV3, ISV4, VVS2 (Thomas and Scott 1993), VVMD5, VVMD7, VVMD25, VVMD27, VVMD28, VVMD32 (Bowers et al. 1996, 1999b) VrZAG62, VrZAG79, VrZAG21, VrZAG112 (Sefc et al. 1999) and VMCNG4b9 (Vitis Microsatellite Consortium), VChr-1b, -2b, -3a, -4a, -5b, -5c, -6a, -7b, -8b, -9a, -10b, -11b, -12a, -13a, -15a, -16a, -17a, -18a, -19a (Cipriani et al. 2008) and VVI-b01, -b23, -b63, -b66, -b94, -f52, -h54, -i51, -m10, -m11, -m25, -n54, -n61, -n94, -o55, -p25b, -p37, -p77, -r09, -s21, -s58, -s63, -u04, -v37, -v61, -v69 (Merdinoglu et al. 2005). A set of chloroplast specific microsatellite loci primers was also employed: ccSSR-5, ccSSR-14, ccSSR-23 (Chung and Staub 2003), cpSSR10 (Weising and Gardner 1999), NTCP8 (Bryan et al. 1999). Chlorotypes definitions are defined following Arroyo-Garcia et al. (2006). The cycling profiles were as reported in Bergamini et al. (2013). PCR reactions were conducted in 10 μl volume containing 25 ng of genomic DNA, 5 pmol of each forward and reverse primer and 5 μl of QIAGEN Fast Cycling PCR Master Mix 2X. Three or more primer pairs were carefully combined to co-amplify in a single reaction and each forward primer was labeled with WellRED dyes, D2-PA (black), D3-PA (green) or D4-PA (blue), at the 5′end. Amplicons were analysed on a CEQ™ 8000 Series Genetic Analysis System, automatically sized using a CEQ DNA Size Standard Kit 400 (Beckman Coulter S.p.A., Milan, Italy), and then visually inspected and manually recorded.

### Data analysis

Amplicons size were rounded, according to the length of the core repeat of each analysed SSR, with an Excel (Microsoft, Redmond, WA) computational sheet. For each considered locus Number of alleles, Expected Heterozygosity, Observed Heterozygosity and Estimated Frequency of Null Alleles were calculated with the aid of the software Identity version 1.0 (Wagner and Sefc 1999) and already reported in Bergamini et al. (2013). Similarly, the same software was used to calculate cumulative Likelihood ratio (LRs) statistics for the detected, putative parent-offspring groups.

### Ampelographic and Ampelometric characterizations

Morphology was recorded in three consecutive years (2009–2011) by means of primary and secondary descriptors, as indicated in the frame of the 2nd edition of the Organisation Internationale de la Vigne et du Vin (OIV) Descriptor List for grape varieties and Vitis species (available: http://www.oiv.int/oiv/files/5%20-%20Publications/5%20-%201%20Publications%20OIV/EN/5-1-9_Liste_descripteurs_2ed_EN.pdf). Ampelometric characterization was performed on 20 leaves sampled at the eighth node for each analyzed variety with the aid of the software SuperAmpelo 2.0 (Comunita`Monastica SS. Pietro e Paolo, Germagno VB, Italy) (Soldavini et al. 2009).

## Results and discussion

### Molecular analysis

South Italian countryside have been explored in the past years to find and recover ancient autochthonous varieties. All recovered accessions were genotyped at 13 microsatellites allowing the unequivocal identification of each analyzed one. The genetic characteristics of our germplasm collection have already been described in a previous paper (Bergamini et al. 2013).

Some peculiar varieties have been characterized at a higher number of microsatellites (Bergamini et al. 2013) to ascertain parental relationships. Overall 107 varieties were evaluated at 54 nuclear microsatellites. Additionally, five plastid microsatellites, commonly used for chloroplast haplotype assaying, were screened. They are used to assign the sex of the two parents in a cross, considering the maternal inheritance of plastid genomes in the *Vitis* genus (Arroyo-García et al. 2006).

**Fig. 1:**
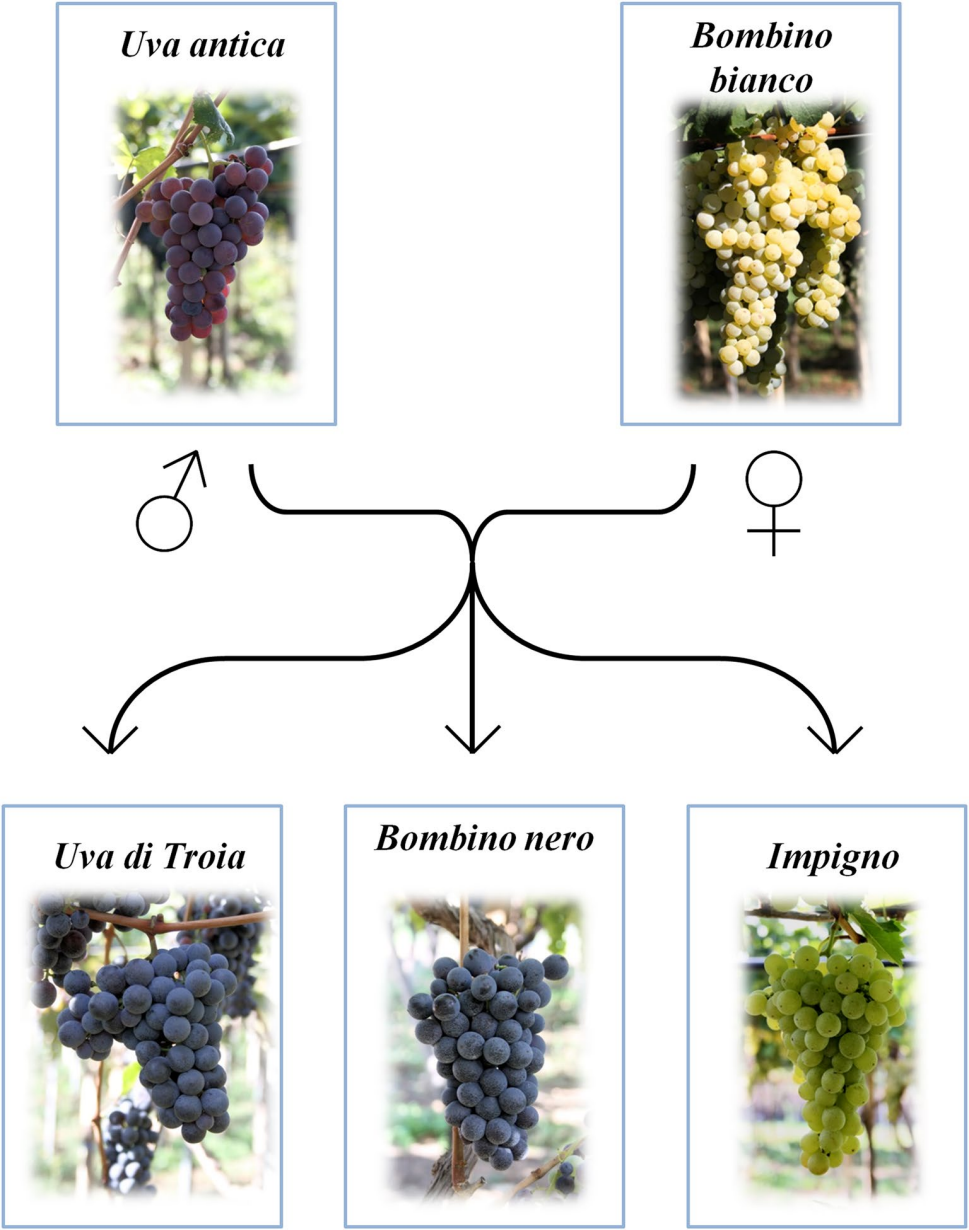
Pedigree of the proposed family

The present study revealed a new family composed of two parents and three siblings. The parents were *Bombino bianco* and *Uva rosa antica*, a previously unknown variety found near Ricigliano, in the Montain townships “Tanagro- Alto e Medio Sele”, province of Salerno. The three siblings were: the renowned *Uva di Troia*, the *Bombino nero* and the *Impigno* (Fig. 1). Values of all 54 nuclear and 5 chloroplast microsatellites are reported in Table 1. As a confirmation, 6 more nuclear microsatellites were additionally analyzed, but only in the five members of the new discovered family, and their values are reported at the bottom of Table 1. Only one of the 60 microsatellites used in this study showed discrepant values for the proposed kinship. VChr-17a was in homozygosis at 189 pb in *Bombino bianco*, and at 179 pb in *Uva rosa antica*: only *Impigno* had heterozygosis 179/189 pb at this locus, while *Uva di Troia* and *Bombino nero* had not the 179 pb allele as expected. As already showed (Bergamini et al. 2013; Gasparro et al. 2013) the VChr-17a microsatellite is strongly affected by null alleles as denoted by the much lower observed heterozygosity frequency (0.308411) compared to the expected heterozygosity frequency (0.501834): the estimated frequency of null alleles is as high as 0.12879 and therefore the *Uva rosa antica* very likely has a null allele and this allele was inherited by *Uva di Troia* and *Bombino nero*.

**Table 1:** Molecular data: The genotype of 60 SSR loci and five chloroplast SSR loci of the analyzed varieties for the proposed kinship and of *Pinot Noir* as reference for size calibration

SSR loci	*Uva rosa antica Quagliano*	*Bombino bianco*	*Uva di Troia*	*Bombino nero*	*Impigno*	*Pinot Noir*
VVS2	133–143	145–151	143–151	133–145	133–145	137–151
VVMD5	225–225	227–231	225–231	225–227	225–227	227–237
VVMD7	238–242	248–252	242–252	238–252	238–248	238–242
VVMD25	239–267	241–263	263–267	239–241	239–241	239–249
VVMD27	178–188	180–194	188–194	178–180	178–194	184–188
VVMD28	249–259	245–249	245–249	249–249	249–249	219–237
VVMD32	240–250	258–272	250–272	240–258	250–272	240–272
VrZAG21	191–205	191– 191	191–191	191–205	191–191	201–207
VrZAG62	185–187	189–199	187–199	185–199	185–189	187–193
VrZAG79	250–254	250–258	250–250	254–258	254–258	240–244
ISV2	166–170	166–170	170–170	166–166	166–170	152–166
ISV3	134–140	140–146	140–140	140–140	140–140	134–146
ISV4	189–199	189–193	189–199	189–189	189–189	171–179
VMCNG4b9	162–164	150–176	164–176	162–176	162–176	158–162
VChr-1b	96–108	100–100	96–100	96–100	100–108	100–108
VChr-2b	116–124	116–124	116–124	116–124	116–124	124–124
VChr-4a	182–199	182–199	199–199	182–182	182–199	182–199
VChr-5b	202–218	202–206	206–218	202–202	202–202	190–190
VChr-5c	104–128	88–120	88–128	104–120	88–104	96–116
VChr-6a	182–186	182–182	182–182	182–186	182–186	182–186
VChr-7b	181–185	181–189	181–185	181–186	181–189	181–189
VChr-9a	90–118	109–115	90–115	90–115	115–118	90–98
VChr-10b	134–134	138–144	134–138	134–144	134–144	138–144
VChr-11b	155–160	157–164	160–164	155–157	155–164	152–164
VChr-12a	137–144	144–144	144–144	144–144	144–144	137–144
VChr-13a	141–157	149–157	157–157	141–149	149–157	157–157
VChr-15a	157–157	149–149	149–157	149–157	149–157	153–157
VChr-16a	110–114	114–114	110–114	110–114	110–114	102–164
VChr-17a	181–**//**	189–189	189–//	189–//	181–189	189–189
VChr-18a	164–168	152–160	160–168	152–164	152–168	164–168
VChr-19a	141–141	138–141	141–141	138–141	138–141	123–138
VVI-b01	298–298	294–298	294–298	294–298	294–298	292–298
VVI-b63	150–154	126–154	126–154	126–154	150–154	148–148
VVI-b94	292–306	284–292	292–306	292–306	284–306	284–296
VVI-f52	262–274	260–260	260–262	260–274	260–262	284–286
VVI-h54	166–168	166–174	166–166	166–174	166–166	164–168
VVI-i51	253–265	263–263	253–263	263–265	263–265	265–267
VVI-m10	357–371	369–379	371–379	357–379	357–369	357–371
VVI-m11	291–301	287–301	287–291	301–301	291–301	291–291
VVI-m25	167–169	177–177	171–177	169–177	167–177	167–187
VVI-n54	100–106	106–112	106–112	106–106	106–106	92–96
VVI-n61	361–375	349–371	349–361	361–371	349–375	371–377
VVI-n94	278–289	286–289	278–289	286–289	289–289	289–289
VVI-o55	141–143	143–147	141–147	141–147	141–147	143–147
VVI-p25b	342–360	340–362	360–362	340–342	360–362	362–362
VVI-p37	150–156	144–148	144–150	148–156	144–150	140–150
VVI-p77	172–182	186–190	172–190	182–186	172–190	180–190
VVI-r09	258–260	258–264	258–264	258–260	258–258	242–248
VVI-s21	284–284	284–290	284–284	284–290	284–284	270–284
VVI-s58	304–308	304–308	308–308	304–308	304–304	294–294
VVI-s63	190–212	190–212	190–212	190–190	190–212	190–190
VVI-u04	182–189	182–191	182–191	182–191	182–189	166–170
VVI-v37	159–167	159–167	159–167	159–167	167–167	149–159
VVI-v69	271–271	257–271	257–271	257–271	257–271	289–291
ccSSR-5	256	255	255	255	255	256
ccSSR-14	201	202	202	202	202	201
ccSSR-23	285	286	286	286	286	285
cpSSR10	110	111	111	111	111	110
NTCP8	250	250	250	250	250	250
VChr-3a	190–190	187–199	190–199	187–190	187–190	202–202
VChr-8b	121–136	103–106	106–121	103–136	103–136	103–139
VrZAG112	231–235	237–245	231–237	231–237	231–237	243–245
VVI-b23	305–323	305–311	305–305	305–305	305–305	309–331
VVI-b66	89–89	89–109	89–109	89–109	89–109	103–109
VVI-v61	169–190	169–192	169–190	169–192	169–190	169–169

The last six loci were analyzed only in the five members of the proposed family. Allele lengths are in bp. ND: not detected

Combined likelihood ratios (LR) for the proposed cross, calculated considering allelic frequencies over 50 loci, are reported in Table 2: LR’s for the proposed cross are 2.90 × 10^36^, 1.69 × 10^41^, 7.87 × 10^30^, and the LR including 95 % upper confidence limits of observed allele frequencies calculated over all loci are respectively 2.92 × 10^25^, 2.32 × 10^29^, 1.36 × 10^21^ (respectively for *Uva di Troia, Bombino nero* and *Impigno;* values calculated on 107 varieties at 50 loci). Even in the most stringent conditions the *Bombino bianco* and *Uva rosa antica* were ranging from 8.63 × 10^4^ to 2.63 × 10^6^ times more probable with 95 % upper confidence than a close relative of one of the two proposed parents was the real parent limit (see values in Table 2 labeled as “rel(2) × (1)” and “(2) × rel(1)”).

**Table 2:** Likelihood ratios: (LRs) for the proposed parentage

*Uva di Troia* = *Bombino bianco* (1) × *Uva rosa antica/Quagliano* (2)					
Combined over all 50 loci in 107 cultivars	X × Y	X × (1)	rel(2) × (1)	(2) × X	(2) × rel(1)
Likelihood ratios including calculated allele frequencies	2.90 × 10^36^	4.49 × 10^25^	5.13 × 10^07^	4.86 × 10^18^	1.74 × 10^06^
Likelihood ratios including 95 % upper confidence limits of observed allele frequencies	2.92 × 10^25^	2.42 × 10^19^	2.50 × 10^06^	1.04 × 10^14^	8.63 × 10^04^
*Bombino nero* = *Bombino bianco* (1) × *Uva rosa antica/Quagliano* (2)					
Combined over all 50 loci in 107 cultivars	X × Y	X × (1)	rel(2) × (1)	(2) × X	(2) × rel(1)
Likelihood ratios including calculated allele frequencies	1.69 × 10^41^	8.45 × 10^25^	5.29 × 10^07^	4.26 × 10^23^	3.90 × 10^07^
Likelihood ratios including 95 % upper confidence limits of observed allele frequencies	2.32 × 10^29^	3.97 × 10^19^	2.63 × 10^06^	1.45 × 10^18^	1.92 × 10^06^
*Impigno* = *Bombino bianco* (1) × *Uva rosa antica/Quagliano* (2)					
Combined over all 50 loci in 107 cultivars	X × Y	X × (1)	rel(2) × (1)	(2) × X	(2) × rel(1)
Likelihood ratios including calculated allele frequencies	7.87 × 10^30^	3.20 × 10^19^	2.79 × 10^06^	3.31 × 10^19^	3.57 × 10^06^
Likelihood ratios including 95 % upper confidence limits of observed allele frequencies	1.36 × 10^21^	3.16 × 10^14^	1.29 × 10^05^	5.85 × 10^14^	1.80 × 10^05^

Likelihood ratios provided in this table are combined over 50 loci

X × Y is the ratio of probability that the proposed parents gave rise to the offspring’s genotype versus the probability that two random individuals give rise to the offspring’s genotype. (Proposed parents) versus (two random cultivars)

X × (1) is the Likelihood ratio for: (Proposed parents) versus (random individual × proposed parent 1)

rel(2) × (1) = (Proposed parents) versus (close relative of proposed parent 2 × proposed parent 1)

(2) × X = (Proposed parents) versus (Proposed parent 2 × random cultivar)

(2) × rel(1) = (Proposed parents) versus (Proposed parent 2 × close relative of proposed parent 1)

Literature search showed that *Impigno*, the less notable of the three siblings, have been previously allocated in a possible kinship: noteworthy, his putative parents were *Bombino bianco*, the same as in our study, and *Quagliano*, a variety cultivated only in some alpine valleys in the north-west of Italy (Cipriani et al. 2010). This coincidence strongly suggested a synonymy between the south Italian *Uva rosa antica* and the north Italian *Quagliano*. To confirm this possibility we compared SSR profiles of *Quagliano* published in the European Vitis Database (available: http://www.eu-vitis.de/index.php) with those we have scored for *Uva rosa antica*, and found identity at all analyzed loci: considering the allelic frequencies of the nine loci used in the European Vitis Database (VVS2, MD5, MD7, MD27, ZAG62, ZAG79, MD25, MD28 and MD32) the combined Probability of Identity (PI) is 6.95438 × 10^10^. Despite the other two siblings, *Uva di Troia* and *Bombino nero,* were included in the analysis of Cipriani et al. (Cipriani et al. 2010), they passed unnoticed because of the high frequency of null alleles occurrence in long-core repeat microsatellites used in that study, one of which likely being VChr-17a. Another earlier study (Zulini et al. 2002) had characterized *Bombino bianco* and *Bombino nero* at six common microsatellites and the two varieties resulted unrelated because of incompatible alleles at the ZAG62 locus but there is an evident mistake in the size call and rounding of values in the *Bombino nero* genotype which were the only ones in the whole list of varieties shifted by just one unit. Recently, a large genotyping study first hypothesize the putative kinship of *Uva di Troia,* albeit using only 20 SSR (Lacombe et al. 2013). Notably, the authors reported that *Quagliano* is not only a synonym of *Uva rosa antica*, but also of *Bouteillan noir*, an old variety cultivated in the Provence region, in the southern France.

Chloroplast microsatellite analysis of the five varieties under study for this family showed two different haplotype for the two parents: type “D” for *Bombino bianco* and type “A” for *Uva rosa antica/Quagliano,* respectively. All three siblings showed the type “D” chloroplast haplotype, thus *Bombino bianco* was the seed-bearer (female parent) and *Uva rosa antica/Quagliano* was the pollen donor (male parent) for all three of them.

### Ampelographic and ampelometric description

**Fig. 2:**
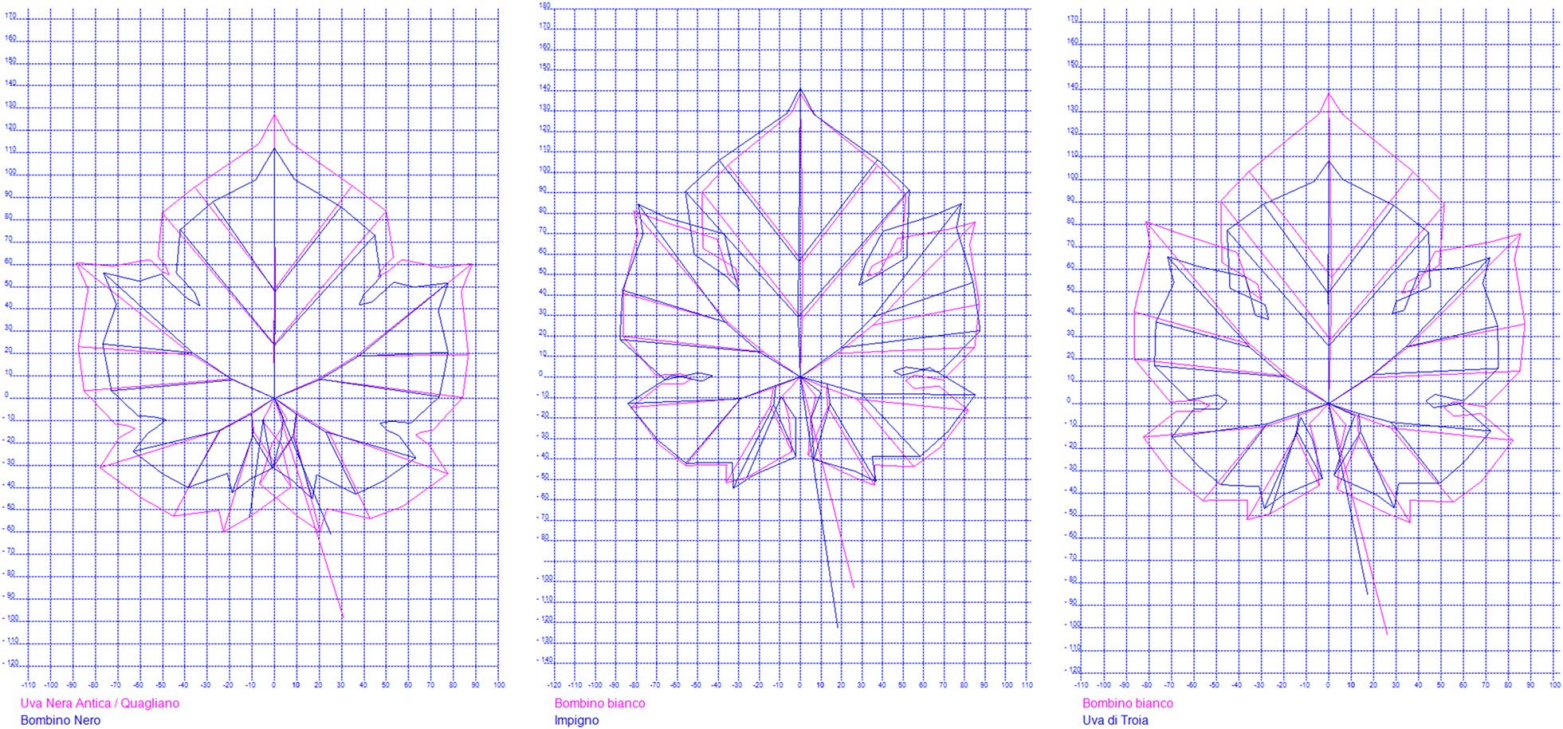
Leaves ampelometric comparison. Ampelometric comparison with the highest Similarity Index between the mean profiles of parental and offspring leaves (respectively *Uva rosa antica/Quagliano* vs *Bombino nero*, *Bombino bianco* vs *Uva di Troia* and *Bombino bianco* vs *Impigno*)

As a reference for other researchers, we report in Table 3 a detailed ampelographic description of the five varieties under study. From the entire ampelographic and ampelometric data set it was possible to perform an analysis of similarity between each parent and its offspring: the software SuperAmpelo (as described in the user manual (http://www.pomologia.it/SuperAmpelo/Documenti.aspx) calculated that the *Bombino nero* has the highest similarity index (63 %) with the parent Uva rosa antica*/Quagliano* while the *Impigno* and *Uva di Troia* show, respectively, a similarity of 70 and 65 % with the parent *Bombino Bianco* (Fig. 2). No other significant morphological similarity were observed between the parents nor the offspring. In spite of very demanding analyzes and generating massive ampelographic and ampelometric data set (in contrary to microsatellites), it was not sufficient to help or contribute revealing of proposed kinship.

**Table 3:** Ampelographic characters: in *Uva rosa antica/Quagliano*, *Bombino bianco*, *Uva di Troia*, *Bombino nero* and *Impigno*

Caracter code and description	*Uva rosa antica/Quagliano*	*Bombino bianco*	*Uva di Troia*	*Bombino nero*	*Impigno*
001 Young shoot: opening of the shoot tip	5	5	5	5	5
003 Young shoot: intensity of anthocyanin coloration on prostrate hairs of the shoot tip	3	7	1	5	1
004 Young shoot: density of prostrate hairs on the shoot tip	1	5	1	5	5
006 Shoot: attitude (before tying)	3	3	3	1	7
007 Shoot: color of the dorsal side of internodes	3	2	3	3	2
008 Shoot: color of the ventral side of internodes	2	2	2	2	3
016 Shoot: number of consecutive tendrils	1	1	1	1	1
051 Young leaf: color of upper side of blade (4th leaf)	1	3	1	1	1
053 Young leaf: density of prostrate hairs between main veins on lower side of blade (4th leaf)	1	5	3	7	1
067 Mature leaf: shape of blade	3	3	2	2	3
068 Mature leaf: number of lobes	3	3	3	2	3
070 Mature leaf: area of anthocyanin coloration of main veins on upper side of blade	1	3	3	1	3
072 Mature leaf: goffering of blade	1	1	1	1	5
074 Mature leaf: profile of blade in cross section	2	1	2	1	1
075 Mature leaf: blistering of upper side of blade	1	1	1	5	1
076 Mature leaf: shape of teeth	4	3	4	5	5
079 Mature leaf: degree of opening/overlapping of petiole sinus	5	5	7	7	7
080 Mature leaf: shape of base of petiole sinus	2	1	1	2	1
081-1 Mature leaf: teeth in the petiole sinus	1	9	1	1	1
081-2 Mature leaf: petiole sinus base limited by vein	1	1	1	1	1
083-2 Mature leaf: teeth in the upper lateral sinuses	1	9	1	1	1
084 Mature leaf: density of prostrate hairs between main veins on lower side of blade	1	5	5	7	3
087 Mature leaf: density of erect hairs on main veins on lower side of blade	7	5	7	1	3
094 Mature leaf: depth of upper lateral sinuses	5	5	5	5	5
202 Bunch: length (peduncle excluded)	5	7	3	3	5
204 Bunch: density	7	3	3	3	7
206 Bunch: length of peduncle of primary bunch	5	3	1	3	3
208 Bunch: shape	1	3	1	1	1
209 Bunch: number of wings of the primary bunch	2	3	2	2	2
220 Berry: length	5	5	5	5	5
221 Berry: width	5	5	5	5	4
223 Berry: shape	2	2	3	2	3
225 Berry: color of skin	5	1	6	6	1
231 Berry: intensity of flesh anthocyanin coloration	1	1	1	1	1

Values are referred to the ‘Descriptor List for grape varieties and Vitis species’ of the Organisation Internationale de la Vigne et du Vin

### Relevant notes on varieties under study

**Fig. 3:**
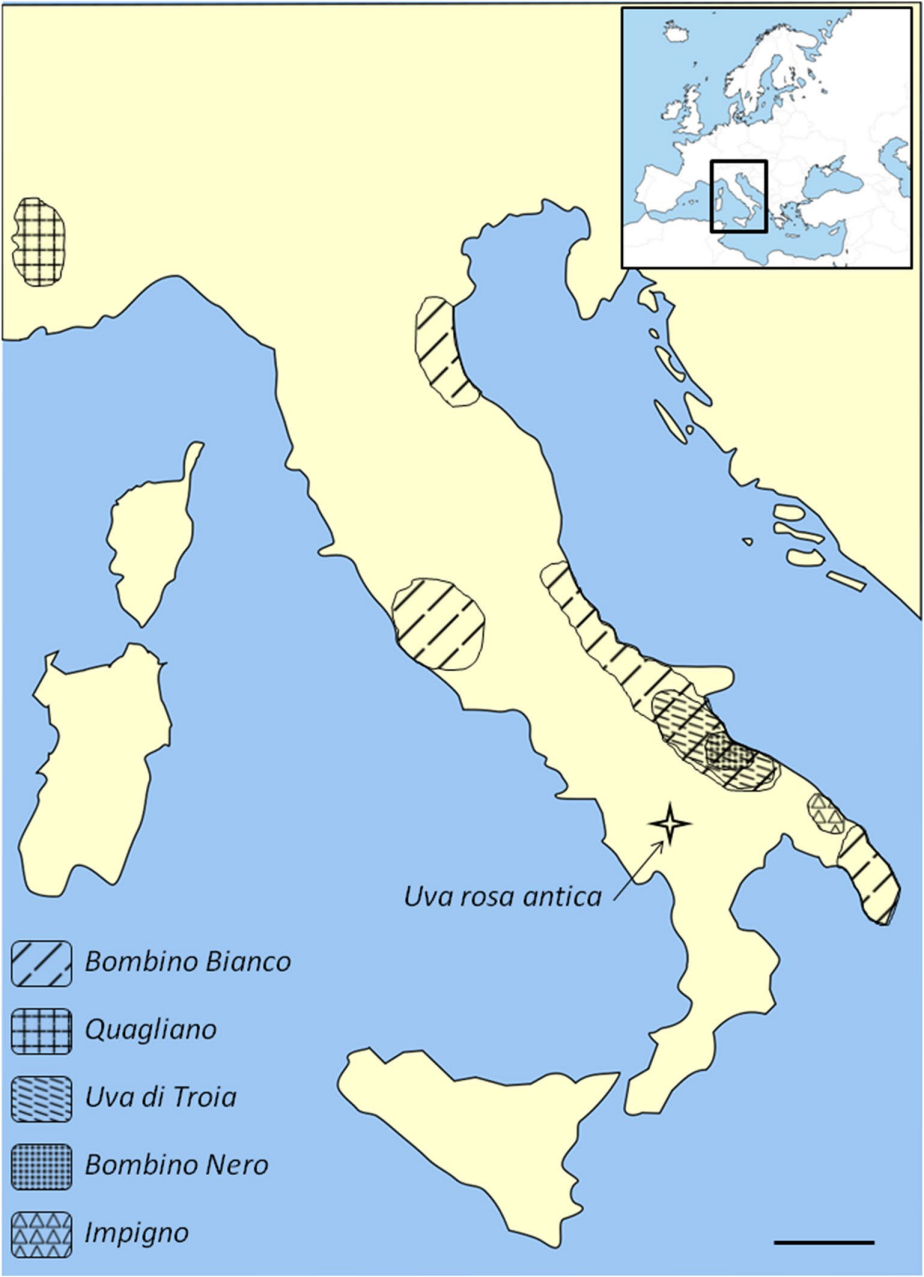
Geographical distribution. This map represent the Italian peninsula; it shows the geographical distribution of areas of certified and protected production of wines (DOC) from the cultivars under study. These varieties are certainly much more spread but there is no other reliable source for tracing their cultivation in other areas. *Scale bar* 100 km

The *Quagliano* is a minor variety, known as being cultivated only in the Piedmont region, north-west of Italy (Fig. 3), exclusively in the Stura, Grand, Maira and Vardita valleys and, until now, its presence was never been reported in other areas of Italy. Its cultivation in those valleys is first documented in an act published in 1739: “Bandi campestri della città di Busca compreso il tenimento detto di Castelreale spettante alla medesima comunità e specialmente li boschi selvatici” (Tenders of Busca rural town including the containment said Castelreale entitled to the same community and especially the wild woods). Slightly earlier, in 1715, is the first documented citation of the *Bouteillan noir*, synonym of *Quagliano* on the other side of the Alps, in the Vaucluse, southern France (Robinson et al. 2013).

The *Bombino bianco* is a variety cultivated in large part of Italy (Fig. 3), having over 70 different known synonymies according to VIVC database, the Vitis International Variety Catalogue (available at www.vivc.de). Its diffusion is probably due to its great productivity, vigor and suitability to different forms of training with high yields per hectare. Its first documented citation dates back to 1825 in Acerbi’s work “*delle viti italiane*” (Acerbi 1825).

The *Uva di Troia* is a variety exclusively cultivated in the south of Italy, especially in the Apulia region and in some part of Campania region (Fig. 3). There are 15 different synonyms reported in the VIVC database for this variety. *Uva di Troia* gives wines that each new vintage are gaining considerable interests, filling the gap of Apulian winemaking, historically devoted to must production. *Uva di Troia* legendary origins and its fascinating name, recalling the ancient poems of Homer, have frequently raised researcher interest. Costacurta and De Gennaro (Costacurta and Germinario 2010), summarizing other studies, set the birth of *Uva di Troia* name in 1875 at the hands of Giovanni Frojo. Before that time it was known only with its synonym Vitigno di Canosa (Bruni 1844). Supposedly, the first indirect citation of *Uva di Troia* dates back to 1791 when the canon Gaetano De Lucretiis reported of a new vines that was spreading in the north Apulian area: he called that variety *Somarello*, a synonym of *Uva di Troia* still in use today in that area (De Lucretiis 1791).

The *Bombino nero* is a variety mostly cultivated in the south of Italy (Fig. 3), there are 5 known synonyms in the VIVC database and, like most of the Apulia varieties, there are no known citation of it preceding the ampelographic descriptions of Di Rovasenda (1877).

The *Impigno* is the last of the three siblings, a minor variety cultivated only in one province of the Apulia region (Fig. 3) and first cited only in 1905 (Del Gaudio and Giusto 1965).

### Historical perspective

Recurrence of families in *Vitis vinifera* L. varieties is not a new feature, indeed it is well known that at least 23 varieties of the northeast of France, some of them are spread internationally by now, are all progeny from repeated crosses starting from early middle age of *Pinot noir* and *Gouais blanc* (Bowers et al. 1999a; Lacombe et al. 2013). Whether the new South Italian family of varieties originated from spontaneous crossing or there were intentionality created, remain a matter of speculation. Althoug science was highly developed in antiquity, the first reported experiments addressing the sexual reproduction in higher plants and assigned the male role to pollen, dates back to the late seventeenth century carried out by the German botanist R.J. Camerarius and brought to an outbreak of experiments in the following centuries up to the celebrated crossings of the Father of Genetics, the Czech monk Gregor Mendel.

In the family we discovered the egg donor parent and the pollen donor one, *Bombino bianco* and *Uva rosa antica/Quagliano* respectively, remain the same for each cultivar of progeny, thus this family could have been originated in a single place and possibly in a single time. A valid hypothesis would also be the common cultivation of these cultivars over long period of time and crossings happened in different places and different time: both cultivars could have been used to be sort of “leading varieties together cultivated” in many vineyard, therefore increasing the chances of repeating the same combination of parents.

Regarding the *Uva rosa antica/Quagliano*, as already mentioned, it was considered a north Italian variety native of the Piedmont valleys, considering the records of its cultivation dating the beginning of the eighteenth century, and only recently shown that it was also contemporary cultivated on the other side of the alps with the synonym *Bouteillan noir*. It is told that the *Quagliano* was used by the Romans to produce wines of the type called *aigleucos*, kept sweet by restraining the fermentation with frequent decanting and immersion of the amphorae into wells (Pliny the Elder, in year 77 AD Historia Naturalia XIV, 11). In the south of Italy the *Uva rosa antica*, synonym of the northern *Quagliano* and *Bouteillan noir*, has been cultivated most likely from the late eighteenth century. In this part of the Italian peninsula documentation is missing regarding cultivated varieties prior to the mid-nineteenth century. Instead, microsatellite analysis revealed its founding role as parent of *Uva di Troia*, *Bombino nero* and *Impigno*. For this purpose it is important to point out that the creation, the evaluation and the spreading of a new variety is a very slow process, taking nowadays more than 30 years: three centuries ago, in an age preceding the New Word pest crisis occurred during the second half of the 1800 s, this procedure could have taken far more than half a century. Furthermore, it could be speculated a role for the prior crisis in the year 1709, this time regarding the climate, the Great Frost or Grand Hiver, the coldest winter of the past 500 years, the most extreme event of the so-called Little Ice Age in the sixteenth to nineteenth centuries (Luterbacher et al. 2004). It caused the loss of many vineyards in northern and central Europe and it could have led to the need of reintroducing varieties spared from the extremely low temperatures in southern areas. This agrees to the *Uva rosa antica/Quagliano* being a late ripening cultivar (Molon 1906), a feature commonly found in varieties cultivated in warmer climates and southern latitudes.

Lastly, in 1877 it was reported by Di Rovasenda in Barletta, south of Italy, the cultivar *Quagliara*, a very similar denomination to *Quagliano*, and if it was the same variety, it could have been its last and only report before being lost (Di Rovasenda 1877).

By combining molecular data and historical documentation, as shown in this study, we are able to propose that the *Uva rosa antica/Quagliano* could actually be an ancient grape variety distributed in several part of the Italian peninsula.

The discovered family relationship joins those already found and it is interesting especially because it relocates the belonging of a wine grapes variety previously considered specific of some alpine valleys in the north-east of Italy and of southern France. This observed genotype reshuffling strengthens the view that most of the modern cultivated grapevine varieties have been obtained over the millennia starting from a limited number of ancestors (This et al. 2006).

## Conclusions

Molecular markers analysis, nuclear and chloroplast microsatellites, has revealed that the *Uva rosa antica/Quagliano* is the progenitor of no less than three southern autochthonous grapevine varieties, among these stands the well known *Uva di Troia. S*ince the family is composed of three autochthonous sibling and two parents of vague origin, the legendary provenance from the city of Troy in Anatolia are certainly disowned because it is very unlikely been introduced in Italy together with the whole family, let alone by an epic hero. The existence of this family moves at least three centuries back the presence of *Uva rosa antica/Quagliano* in the south of Italy. Additionally, this family point out the genetic proximity or limited genetic diversity of modern cultivated grapevine. The preservation of the existing biodiversity and the recovery of ancient cultivars is a key point for the scientific research, whereas the molecular marker analysis can allow to clarify the evolutionary history of the current varieties especially these varieties that have major features in terms of oenological quality: reconstructing their history could be useful to understand how these favorable characteristics are genetically determined in order to plan later improvements.
